# Perspectives of laypeople towards the morphological characteristics of a youthful smile on frontal view: A computerized simulated photographic evaluation

**DOI:** 10.1016/j.jobcr.2025.03.008

**Published:** 2025-03-22

**Authors:** Marzieh Mazhari, Sanaz Zemanatkheiri, Mehrnaz Moradinejad

**Affiliations:** a Department of Orthodontics, School of Dentistry, Ahvaz Jundishapur University of Medical Sciences, Ahvaz, Iran; b Department of Dentistry, School of Dental, Ahvaz University of Medical Sciences, Ahvaz, Iran

**Keywords:** Youthful smile, Incisor display, Vertical step, Smile arch, Buccal corridor, Frontal view

## Abstract

**Background:**

The desire for a youthful smile is not limited by age, as individuals seek it throughout their lifetime. Aging affects the components of a smile; accordingly, this study assessed the perspectives of laypeople towards the morphological traits of a youthful smile on the frontal view. In this psychometric study**,** a 26-year-old female with a normal face, normal occlusion, teeth alignment, and no previous cosmetic surgery was employed as the photo model; sixteen smile images were obtained via Adobe Photoshop by varying incisal display, vertical step between maxillary central and lateral incisor, smile arch form, and buccal corridor size. The prepared photos were given to the layperson participants using an online questionnaire for age estimation.

**Results:**

A total of 195 laypersons participated in the study. The results revealed that a 2 mm reduction, 4 mm increase, and 4 mm reduction in incisor display would increase the age estimation (P-Value<0.001). Futher, 1 mm reduction, a 0.5 mm reduction, and 0.5 mm increase in vertical step would contribute to overestimation of the age (P-Value<0.001). Additionally, the smile arc's reversed, straight, and moderate convexity increased the age estimation (P-Value<0.001). Regarding buccal corridor size, 1- and 2-mm reduction plus 2-mm increase resulted in age estimates significantly older than the original image (P-Value<0.001).

**Conclusion:**

Perceptions of youthfulness are influenced by morphological traits such as incisor display, vertical step between central and lateral, smile arch form, and buccal corridor size.

## 1 Introduction

Facial and dental beauty plays a significant role in life quality, affecting how people are viewed and treated in major social issues. Among the aesthetic criteria, the smile has attracted the great attention of researchers. An aesthetic smile significantly influences the subjects' spirit, social acceptance, and self-confidence.^1^ A smile is formed by raising the corner of the mouth and is usually used to show pleasure, happiness, or confidence. The face and smile beauty attractiveness are strongly interrelated to each other, and a smile is the most essential feature in the facial attractiveness after the eyes. The desire for a young and attractive face is not limited by age. Due to aging, the smile's anatomy changes, and facial sagging becomes evident.

Modifying a smile is one of the main targets in cosmetic dentistry treatments to make people look younger. This change can alter the appearance and alignment of teeth as well as gingival tissues. Several factors influence smile attractiveness, including smile arch, gingival exposure, buccal corridor size, the coincidence of upper and lower midline, tooth proportions, teeth color, and cant of occlusal plane.

The size of the buccal corridor, which refers to the space between the maxillary teeth' buccal surface and the mouth's corner, is one of the controversial aspects of smile attractiveness.^2^^,^^3^ There are different opinions about the effect of buccal corridor size on smile attractiveness. A constricted maxillary arch, lingually–inclined maxillary molars, and tooth extraction treatment have been proposed as the causes of buccal corridor widening.^4^^,^^5^

The ideal smile arc is a complex issue in orthodontics. A study found that the smile arc is the most important factor in smile attractiveness.^6^ Several types of smile arcs influence the attractiveness of a smile.^7^ On the other hand, there is a disagreement between dental experts about the criteria for smile arc esthetics. Understanding the optimal smile arc and its related factors is useful in improving the attractiveness of orthodontists. The criteria for aesthetics differ between regions; in South Asia, the average smile line, consonant smile arc, and cuspid-type smiles are more frequent. In this regard, several studies have evaluated the factors influencing the perception of an aesthetic smile and their applicability in cosmetic dentistry. In this regard, the present study's primary aim is to examine laypeople's perspectives towards the morphological characteristics of a youthful smile concerning the resulting changes in smile arc, buccal corridor size, incisal show, and vertical step between maxillary central and lateral incisor.

## 2 Material and method

The present study was a prospective study on 195 raters towards 19 smile images with different morphological traits. The participants over 18 were randomly selected from the general population. The inclusion and exclusion criteria were considered as follows: lack of knowledge of smile parameters meaning that they were not employed in related professions such as dentistry, medicine, painting, hairdressing, etc. Written informed consent was obtained from raters and the photo model.

## 3 Sample size

The laypersons were considered as the judges (raters), who were >18 years old and had no dental or medical background, and their professions were not related to art and aesthetics. The sample size was calculated based on the study of Desai S et al. and the following formulate.^8^ Considering α = 0.05, s = 2.12, and d = 0.3, the total sample size was calculated for 195 subjects.

$$n=\frac{{\left(Z_{1-\frac{\alpha }{2}}\right)}^{2}\left(s^{2}\right)}{{\left(d\right)}^{2}}$$

## 4 Original photographs

A frontal view photograph was obtained from the photo model (26 years old) in social smile mode (Canon, USM IS 135-18 90 D). The photograph was obtained in the presence of an orthodontist in Natural Head Position (NHP). The female photo model had the following characteristics:

• No history of facial cosmetic surgery or procedures
• No history of dental cosmetics procedures (such as veneer or laminate)
• Normal overjet and overbite
• No missing teeth or hypodontia
• Ideal alignment of maxillary teeth
• Iranian face without the characteristics of Western faces, such as colored eyes and golden hair
• Ideal incisal and gingival show a smile

## 5 Perceptual metric image sets with controlled variable morphologies

**Fig. 1:**
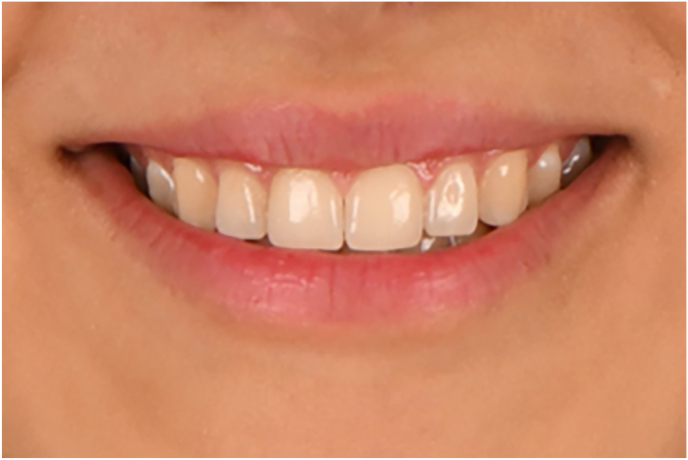
The original photo of the model.

The original photo was considered as a control (Fig. 1). The incisor display, buccal corridor size, smile arc, and step between the maxillary central and lateral incisor (on both sides) were altered by Adobe Photoshop CC (2021, v22.5.9.1101).

**Fig. 2:**
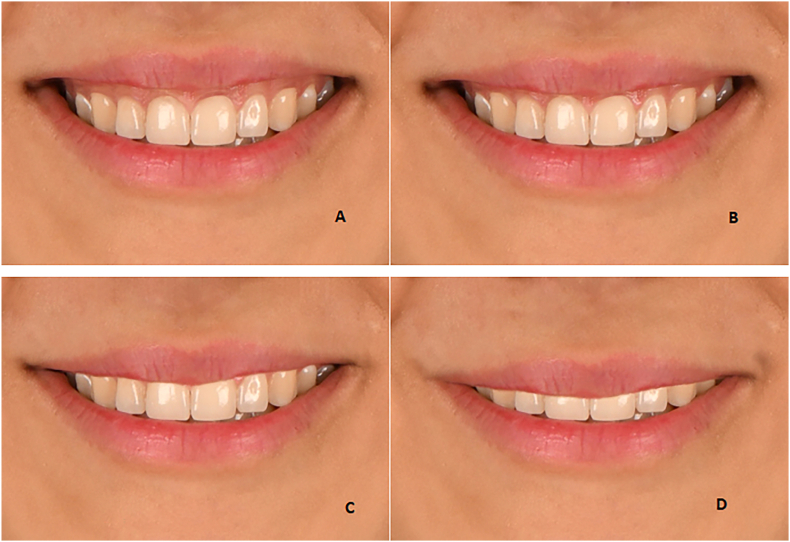
The incisor displays **(A)** a 4-mm increase, **(B)** a 2-mm increase, **(C)** a 2-mm decrease, and **(D)** a 4-mm decrease.
⁃ 2-mm increase (Fig. 2B)
⁃ 2-mm decrease (Fig. 2C)
⁃ 4-mm decrease (Fig. 2D).

The detailed alterations in different traits were as follows:

⁃ 4-mm increase (Fig. 2A)

## 6 Buccal corridor

**Fig. 3:**
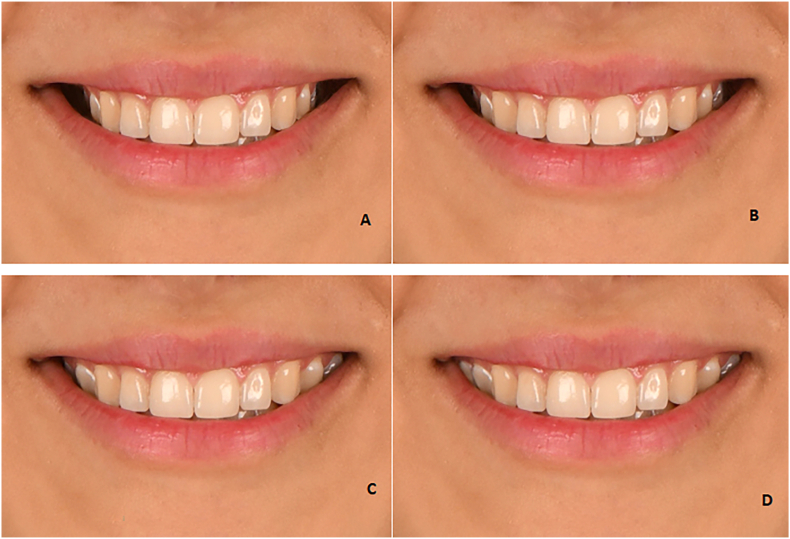
The buccal corridor displays **(A):** 2-mm increase, **(B):** 1-mm increase, **(C):** 1-mm decrease, and **(D):** 2-mm decrease.
⁃ 1-mm increase in buccal corridor (Fig. 3B)
⁃ 1-mm decrease in buccal corridor (Fig. 3C)
⁃ 2-mm decrease in buccal corridor (Fig. 3D).

Four photos with the following buccal corridor were prepared:

⁃ 2-mm increase in buccal corridor (Fig. 3A)

## 7 Smile arc

**Fig. 4:**
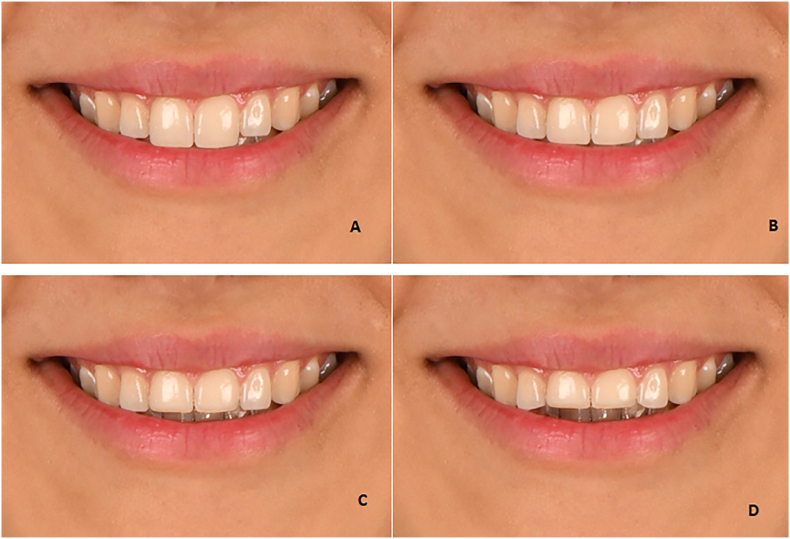
The **(A)** severe smile arc, **(B)** medium smile arc, **(C)** flat smile arc, **(D)** reverse smile arc.

Four photographs with four types of smile arcs, including severe curve (Fig. 4A), medium curve (Fig. 4B), flat (Fig. 4C), and reverse (Fig. 4D), were prepared.

**Fig. 5:**
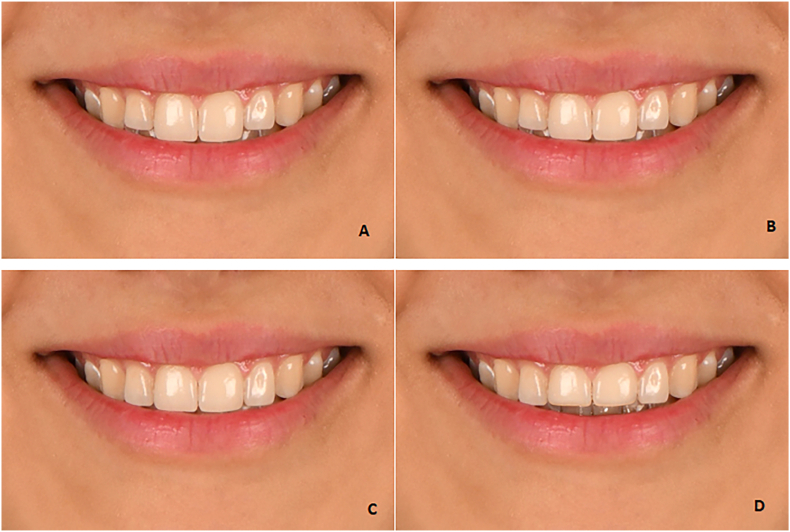
Vertical step between the maxillary central and incisor; 1-mm increase (Fig. 5A), 0.5-mm increase (Fig. 5B), 0.5-mm decrease (Fig. 5C), 1-mm decrease (Fig. 5D).

## 8 Vertical step between maxillary central and lateral incisor

Four photos with the following vertical step between the maxillary central and lateral incisor were prepared:

⁃ 1-mm increase (Fig. 5A),
⁃ 0.5-mm increase (Fig. 5B)
⁃ 0.5-mm decrease (Fig. 5C)
⁃ 1-mm decrease (Fig. 5D).

## 9 Questionnaire

The prepared photos were given to the layperson participants using an online questionnaire for age estimation. The 16 prepared photos with the original image were randomly inserted into the questionnaire. Two copies of the original photo were placed to evaluate the reliability. Each slide of the questionnaire included one image and a question, and for all the questions, six options regarding age were considered as follows:

• 18–21 years
• 22–25 years
• 26–29 years
• 30–33 years
• 34–37 years
• ≥38 years

The participants were asked to choose an option for each image representing the estimated age. The participants had 20 s to view each question. The option to return to the previous question was disabled, so the participants could not compare the images. No instructions were given to the participants, so they would not focus on a particular part of the smile and judge the photos based on their opinions as well as perceptions. Along with the first three questions of the questionnaire (gender, age, education, and job), the maximum time to complete the online questionnaire was estimated to be 7 min.

## 10 Statistical analysis

All data were analyzed using SPSS software (IBM SPSS Statistics V26, SPSS Inc., Chicago, IL, USA). The Kolmogorov-Smirnov test assessed the normality of data. Frequency descriptive methods would be discussed, including frequency distribution tables, charts, central indices, and appropriate dispersion to describe the variables. The T-test determines the relationship between qualitative and quantitative variables. The level of significance was set as 0.05.

## 11 Results

One hundred ninety-five laypersons, including 141 females (72.3 %) and 54 males (27.7 %), participated in the present study. The mean age of subjects was 28.18 ± 8.21 years old. Bachelor's degree was the most (37.4 %) frequent education level among participants (Table 1).

**Table 1:** The demographic information of participants.

Variable		Count	Frequency (%)
Gender	Male	54	27.7
	Female	141	72.3
Education	Under diploma	18	9.2
	Diploma	54	27.7
	Bachelor's degree	73	37.4
	Master's degree	40	20.5
	PhD	10	5.1

To assess the results' reliability, raters evaluated the original photo three times. The results revealed that in the first original photo, 61 laypersons (31.4 %); in the second photo, 61 subjects (31.3 %); and in the third photo, 59 (30.3 %) of participants estimated the age as 26–29 years old (Table 2).

**Table 2:** The results of the reliability.

Age (year)	First original photo (%)	Second original photo (%)	Third original photo (%)
18–21	28 (14.4)	24 (12.5)	22 (11.3)
22–25	61 (31.4)	44 (22.9)	48 (24.6)
26–29	61 (31.4)	60 (31.3)	59 (30.3)
30–33	29 (14.9)	39 (20.3)	41 (21)
34–37	11 (5.7)	19 (9.9)	23 (11.8)
≥38	4 (2.1)	6 (3.1)	2 (1)
Mean ± SD	26.37 ± 4.67	27.54 ± 5.05	27.51 ± 4.85

## 12 Impact of the incisor display

In photos presenting 2- and 4-mm increase plus 2 mm decrease in incisal display, the most estimated age was 26–29 years (33.3 %), (27.7 %), and (30.1 %), respectively (Table 3). For the photos presenting a 4 mm decrease, the most estimated age was 30–33 years (Table 3).

**Table 3:** The frequency of age estimation based on different incisal display.

Age (year)	2 mm increase (%)	4 mm increase (%)	2 mm decrease (%)	4 mm decrease (%)
18–21	27 (14.3)	26 (13.3)	16 (8.3)	15 (9.7)
22–25	55 (29.1)	38 (19.5)	40 (20.7)	37 (19.4)
26–29	63 (33.3)	54 (27.7)	58 (30.1)	37 (19.4)
30–33	33 (17.5)	46 (23.6)	45 (23.3)	45 (23.6)
34–37	9 (4.8)	22 (11.3)	26 (13.5)	32 (16.8)
≥38	2 (1.1)	9 (4.6)	8 (4.1)	25 (13.1)

In comparison to the original photo, the age was significantly overestimated in photos of 4-mm reduction (29.88 ± 5.86), followed by 2-mm increase (28.49 ± 5.05) and 4-mm increase (28.03 ± 5.34) in incisal display (P-Value <0.001) (Table 4). The difference was not significant regarding the 2-mm reduction (P-Value = 0.965).

**Table 4:** Comparing the age estimation based on the incisal display.

Incisal display	Mean ± SD	Basic	P-Value
2 mm reduction	26.39 ± 4.46	26.37	0.965
4 mm reduction	29.88 ± 5.86		<0.001
2 mm increase	28.49 ± 5.05		<0.001
4 mm increase	28.03 ± 5.34		<0.001

## 13 Impact of the vertical step between maxillary central and lateral incisor

The most prevalent lowest estimated age (22–25 years) was observed in a 1-mm increase (39.1 %), while in 1- (26.4 %) and 0.5-mm (32.3 %) step reduction and 0.5-mm (24.4 %) increase, the age 26–29 was more prevalent (Table 5).

**Table 5:** The frequency of age estimation based on the vertical step.

Age (year)	1 mm increase (%)	0.5 mm increase (%)	1 mm decrease (%)	0.5 mm decrease (%)
18–21	22 (11.5)	28 (14.5)	20 (10.4)	19 (10.1)
22–25	75 (39.1)	41 (21.2)	34 (17.6)	48 (25.4)
26–29	40 (20.8)	47 (24.4)	51 (26.4)	61 (32.3)
30–33	36 (18.8)	47 (24.4)	39 (20.2)	41 (21.7)
34–37	4 (7.3)	24 (12.4)	35 (18.1)	10 (5.3)
≥38	5 (2.6)	6 (3.1)	14 (7.3)	10 (5.3)

Further analysis revealed that in comparison with the original photo, the age was overestimated by a 1-mm increase (29.06 ± 5.62), 0.5-mm increase (27.81 ± 5.35), and 0.5-mm reduction (27.57 ± 4.94) in vertical step between maxillary central and lateral incisor (P-Value <0.001) (Table 6). However, no significant difference was observed for 1-mm reduction (P-Value = 0.441).

**Table 6:** Comparing the age estimation based on the vertical step.

Vertical step	Mean ± SD	Basic	P-Value
1 mm reduction	26.65 ± 4.90	26.38	0.441
0.5 mm reduction	27.57 ± 4.94		<0.001
1 mm increase	29.06 ± 5.62		<0.001
0.5 mm increase	27.81 ± 5.35		<0.001

## 14 Impact of smile arc

Considering the smile arc, the most estimated age was 26–29 years (Table 7). According to the participants, the severe curve in smile arcs (27.4 %) was younger (22–25 years); in contrast, the reverse (24.1 %), flat (24.1 %), and medium (30.9 %) smile arcs were estimated older (26–29 years) (Table 7).

**Table 7:** The frequency of age estimation based on the smile arc.

Age (year)	Reverse smile arc (%)	Severe smile arc (%)	Flat smile arc (%)	Medium smile arc (%)
18–21	20 (10.5)	24 (12.6)	22 (11.3)	25 (13.1)
22–25	41 (21.5)	52 (27.4)	41 (21)	44 (23)
26–29	46 (24.1)	56 (29.5)	47 (24.1)	59 (30.9)
30–33	36 (18.8)	40 (21.1)	44 (22.6)	41 (21.5)
34–37	30 (15.7)	16 (8.4)	29 (14.9)	15 (7.9)
≥38	18 (9.4)	2 (1.1)	12 (6.2)	7 (3.7)

Our data indicated that the age was overestimated in the reverse smile arc (28.89 ± 8.82), followed by flat (28.55 ± 5.55) and medium curve smile arcs (27.44 ± 5.04) (P-Value <0.001) (Table 8). The difference was not significant regarding the severe curve smile arc (P-Value = 0.06).

**Table 8:** Comparing the age estimation based on the smile arc.

Smile arc	Mean ± SD	Basic	P-Value
Reverse	28.89 ± 8.82	26.38	<0.001
Severe	27.03 ± 4.74		0.06
Flat	28.55 ± 5.55		<0.001
Medium	27.44 ± 5.04		<0.001

## 15 Impact of buccal corridors

The youngest estimated age was 22–25 years, which was observed in 1 mm reduction (26.9 %), while in 1 (27.3 %) and 2 (21.8 %) mm increase, and 2 mm reduction (31.3 %), the youngest estimated age was 26–29 years (Table 9).

**Table 9:** The frequency of age estimation based on the buccal corridor size.

Age (year)	1 mm increase	2 mm increase	1 mm reduction	2 mm reduction
18–21	31 (16)	31 (16.1)	19 (9.8)	25 (13)
22–25	52 (26.8)	40 (20.7)	52 (26.9)	48 (25)
26–29	53 (27.3)	42 (21.8)	50 (25.9)	60 (31.3)
30–33	45 (23.2)	40 (20.7)	44 (22.8)	40 (20.8)
34–37	9 (4.6)	21 (10.9)	20 (10.4)	15 (7.8)
≥38	4 (2.1)	19 (9.8)	8 (4.1)	4 (2.1)

The final analysis demonstrated that in comparison with the original photo, 2-mm increase (27.85 ± 5.13), 1-mm increase (27.85 ± 5.13), and 2-mm reduction in buccal corridors (27.85 ± 5.13), respectively (P-Value <0.05) (Table 10) caused the participants to wrongly estimate the model's age as 26–29 years. However, no significant difference was found in the 1-mm reduction of buccal corridor (P-Value = 0.380) (Table 10).

**Table 10:** Comparing the age estimation based on the buccal corridor.

Buccal corridor	Mean ± SD	Basic	P-Value
1 mm reduction	26.68 ± 4.83	26.38	0.380
2 mm reduction	27.15 ± 4.84		0.027
1 mm increase	28.21 ± 6.04		<0.001
2 mm increase	27.85 ± 5.13		<0.001

## 16 Discussion

Smile is an essential element of nonverbal social interaction. Maintaining the dynamics of the smile is of importance in making a person look attractive. Understanding the effective ways to make a young and more attractive smile leads to increased self-confidence, especially in older people. Due to the aging of facial muscles, the signal strength, conveying sense, and facial expression are reduced.

The most critical factor in smile esthetics is the smile arc. During orthodontic treatment, the smile arc is the item that can easily be flattened following other treatment objectives.^9^ Our study revealed that from a layperson's point of view, the severe convexity of the smile arc makes the smile more youthful compared to a flat and reverse one. As the smile arc is defined as the relationship between the upper teeth and the lower lip, any change in these components leads to an altered smile arc. It has been demonstrated that the concave curvature of the lower lip is not always influenced by age.^10^

The buccal corridor is the most challenging factor in smile attractiveness since there is no consensus on its ideal size.^11^, ^12^, ^13^ Findings indicate that a wide buccal corridor reduces the beauty and youthfulness of a person's face.^14^ Aging leads to a drop in perioral soft tissue and reduced buccal corridor size^15^^,^^16^ Our findings demonstrated that any increase in the buccal corridor size and a 2-mm reduction lead to age overestimation, where the younger estimation is related to a 1-mm reduction. Rajeev et al. found that general dentists and laypeople preferred 2 % and 10 % buccal corridor widths for smile attractiveness.^17^ In agreement with this finding, Niknam and Oz et al. have shown that narrow and medium buccal corridor widths (0 and 12 %) are preferred as attractive smiles.^18^^,^^19^

The maxillary incisal display plays a paramount role in smile attractiveness since youthful smiles show more maxillary incisors while aged smiles tend to reveal less.^20^ The present findings indicated that age was overestimated in photos of 4-mm reduction and 2-mm and 4-mm increase in incisal display. This finding was in line with Awad et al., who indicated that the maxillary incisal display was inversely proportional to age.^21^ Since incisal position can be dramatically altered by orthodontic or orthosurgery treatment, clinicians should take care in bracket positioning in the aesthetic zone, as maxillary central incisors should appear more extruded than intruded to guarantee a youthful smile.^22^ In addition to age, maxillary incisor positioning is influenced by so many variables, including gender, tooth anatomy, mandibular lateral excursive and protrusive movements, over-bite, etc..^23^ Therefore, the decision on the most attractive, stable, and functional position of the upper incisors should be carefully discussed with patients.

The vertical relationship between the edges of the central and lateral incisors is a major criterion to be considered in the treatment plan, bonding, orthodontic finishing, and detailing.^24^ The present findings revealed that 0.5- and 1-mm increase as well as 0.5 mm reduction in vertical step between maxillary central and lateral incisor would lead to age overestimation. The results of Machado et al. demonstrated that the impact of the incisal edge relationship on smile esthetics is more important than the gingival display; so, the proper positioning of the incisal edges is preferred to gingival margins and gingival display.^25^ Their results revealed that 1.5 mm of step between the central to lateral incisors created the highest-rated smile; in contrast, no step between the central and laterals has the lowest attractiveness.^25^ In a study conducted by King et al., the lateral incisors being 0.6 mm above the incisal plane were preferred.^26^ The findings of Daou et al. indicated that 1- and 0-mm steps between the lateral and the central incisors are most attractive vertical from the point of view of orthodontists and laypersons, respectively.^27^ The results of the present study also demonstrated that an increase in vertical steps is not considered youthful and attractive from a layperson's point of view. This result is remarkable and could explain the tendency of patients to prefer well-aligned teeth, all at the same level.

In order to enhance focus on dental alterations and reduce the distraction of judges, close-up smile photos were taken and rated. Some studies have shown that the aesthetic impact of smile visualization is more significant in a dental view compared with a full facial view.^28^^,^^29^

In the current investigation, by eliminating the nose and chin, the bias of participants' answers was reduced.^6^^,^^25^^,^^30^^,^^31^ On the other hand, no significant difference was found between close-up and full-face view smile assessment.^22^

The participants in this survey were just laypersons, whereas comparing them with orthodontics would give us further insights. Due to their science regarding aesthetic criteria, orthodontists have stricter opinions than laypersons.^6^^,^^9^^,^^24^^,^^32^, ^33^, ^34^, ^35^ Additionally, due to the subjectivity of smile aesthetic evaluation, caution should be exercised when making them applicable to aesthetic improvement.^32^ In this regard, communication with the patient during the treatment is essential for better outcomes.

Younger and more attractive smiles directly influence self-confidence. The laypeople's view is very useful in fulfilling this confidence. The present study, by providing valuable intricate details, can be helpful for professionals and orthodontists to understand what younger and more appropriate smile would look like.

Evaluating a man model would simultaneously give us valuable information which was not studied and was the main limitation of the present investigation. The other limitation of our study was the lack of data about laypersons' education degrees, which can cause bias in the results.

## 17 Conclusion

According to the present study, the most influential parameter in creating an old smile was 4-mm reduction of incisor display. Other parameters including 2- and 4-mm increase of incisor display, 0.5-and 1-mm increase, plus 0.5-mm reduction in vertical step between the maxillary central and lateral incisor, reverse, flat, and medium convexity of smile arc, 1-and 2-mm increase as well as 2-mm reduction in buccal corridor would also significantly make the smile older.

## Patient's/Guardian's consent

Written informed consent was obtained from raters and the photo model.

## Ethics approval

The current study is based on the ethical committee of Ahvaz Jundishapur University of Medical Sciences (IR.AJUMS.REC.1402.114).

## Consent for publication

Written informed consent was obtained from both patients to publish this case report and accompanying images. A copy of the written consent is available for review by the Editor-in-Chief of this journal on request.

## Availability of data and materials

The current study has generated and/or analyzed datasets available in the [PubMed, Web of Science, Scopus, EM Base] repository.

## Ethics approval

The current study is based on the ethical committee of Ahvaz Jundishapur University of Medical Sciences (IR.AJUMS.REC.1402.114).

## Declaration of generative AI in scientific writing

During the preparation of this work the authors not used any AI tool/service.

## Funding

None.

## Declaration of competing interest

The authors declare that they have no known competing financial interests or personal relationships that could have appeared to influence the work reported in this paper.

## List of abbreviations

No abbreviations.
